# Seldinger versus Trocar technique for percutaneous cholecystostomy: impact of access route on procedural time and post-procedural pain

**DOI:** 10.1007/s00261-026-05585-z

**Published:** 2026-06-23

**Authors:** Natthaphong Nimitrungtawee, Warunya Thawang, Phichayut Phinyo, Nop Khongthon, Tanop Srisuwan, Nakarin Inmutto, Attaporn Jantarangkoon

**Affiliations:** 1 Diagnostic Radiology Unit, Department of Radiology, Faculty of Medicine, Chiang Mai University, 50200 Chiang Mai, Thailand; 2 Department of Biomedical Informatics and Clinical Epidemiology (BioCE), Faculty of Medicine, Chiang Mai University, 50200 Chiang Mai, Thailand; 3 Musculoskeletal Science and Translational Research (MSTR), Faculty of Medicine, Chiang Mai University, 50200 Chiang Mai, Thailand

**Keywords:** Cholecystostomy, Cholecystitis, Radiology, Interventional, Drainage, Pain measurement

## Abstract

**Objective:**

To compare procedural time (primary outcome) and technical success, clinical success, post-procedural pain and safety outcomes between the Seldinger and Trocar techniques for percutaneous cholecystostomy (PC) in high-risk patients with acute cholecystitis.

**Materials and methods:**

This retrospective cohort study included 169 consecutive patients (Seldinger: *n* = 130; Trocar: *n* = 39) who underwent ultrasound-guided PC between January 2020 and August 2024. The primary outcome was procedural time. Secondary outcomes were technical and clinical success, post-procedural pain (defined as the requirement for strong [WHO Step-3] opioid analgesics), adverse events graded per the Society of Interventional Radiology (SIR) Adverse Event Classification, and 30-day mortality. Multivariable regression with marginalization was used to estimate adjusted associations between technique and outcome, adjusting for disease severity (TG18 grade), comorbidities, gallbladder morphology, and anatomical approach (transhepatic vs. transperitoneal). Inverse-probability-of-treatment weighting and confounder-score adjustment were performed as sensitivity analyses.

**Results:**

The Trocar technique was significantly faster (median procedural time 81 s [IQR 69–99] vs. 246 s [IQR 199–399]; adjusted mean difference −212.78 s; 95% CI −314.68, − 110.88; *p* < 0.001). Both techniques achieved high technical (99.2% vs. 100%) and clinical success rates (98.5% vs. 94.9%). Overall opioid requirement did not differ between techniques (adjusted OR 1.01; *p* = 0.97). A significant interaction (effect modification) was observed between technique and access route: in the transperitoneal subgroup the Seldinger technique was associated with a 7-fold higher risk of severe pain than the Trocar technique (adjusted OR 7.36; 95% CI: 1.48, 36.65; *p* = 0.015), whereas no difference was found in the transhepatic subgroup. The 30-day crude mortality rate was higher in the Trocar group (23.1% vs. 6.2%); cause-specific adjudication suggested that the excess deaths were attributable to non-biliary sepsis and underlying comorbidities rather than procedure-related events, although residual selection bias toward use of the faster technique in unstable patients cannot be excluded.

**Conclusion:**

The Trocar technique is a time-efficient alternative to the Seldinger technique for percutaneous cholecystostomy. Our findings suggest that, when transperitoneal access is anatomically unavoidable, the Trocar technique may be preferred to reduce procedural time and severe post-procedural pain. Confirmation in larger prospective studies is warranted.

**Graphical abstract:**

**Figure:**
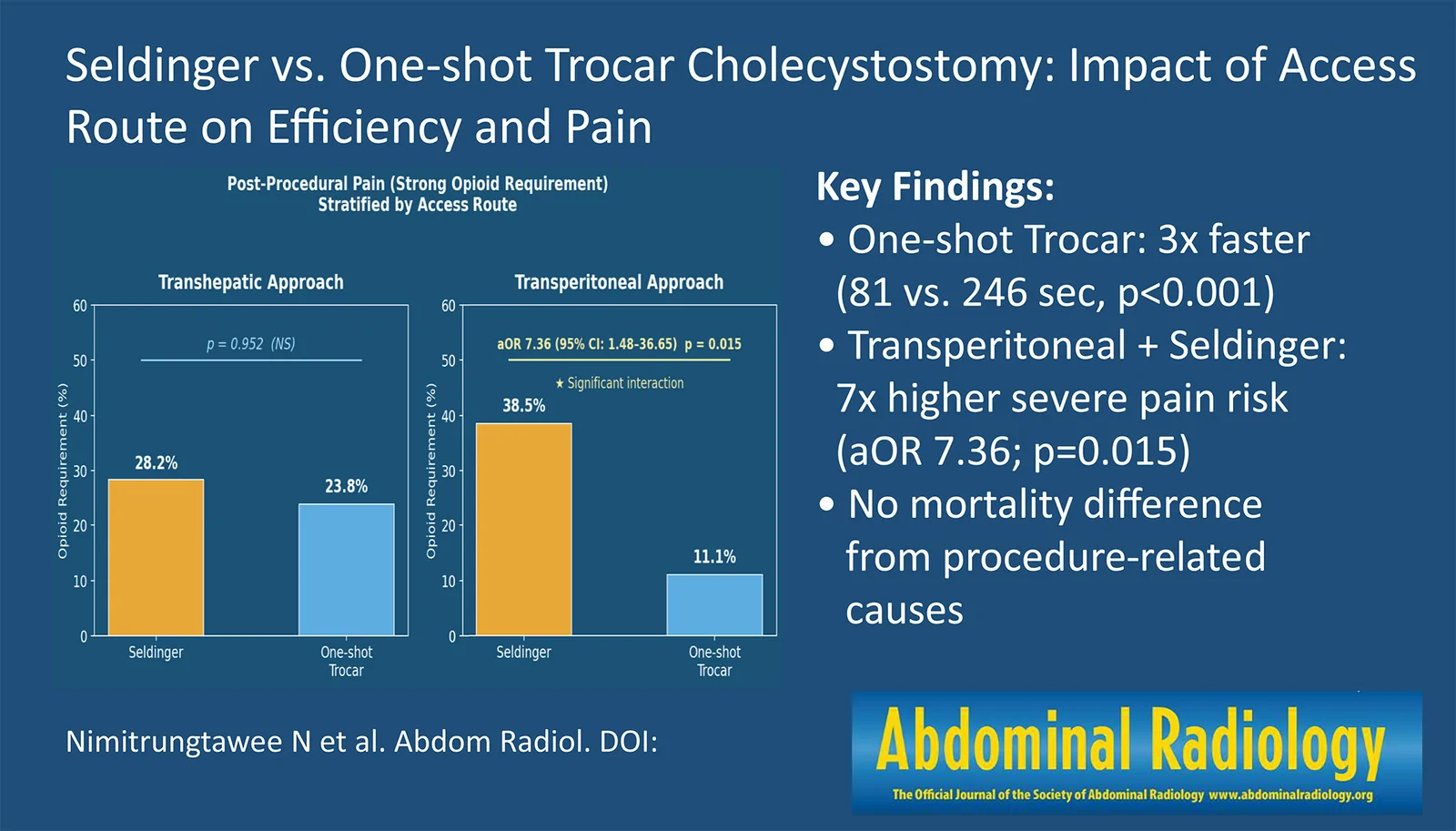

## Introduction

Acute cholecystitis (AC) is a common cause of emergency hospital admission, with laparoscopic cholecystectomy as the gold-standard treatment. In patients deemed unfit for surgery because of severe inflammation, severe sepsis, or significant comorbidity, percutaneous cholecystostomy (PC) provides effective gallbladder decompression and source control [1–5]. Reported technical success exceeds 95% and clinical response approaches 86%, and a randomized trial in high-risk patients has demonstrated benefit over conservative management followed by delayed cholecystectomy [6–10].

Regarding technical aspects, PC encompasses diverse insertion techniques and anatomical approaches. The procedure is primarily performed using one of two techniques: the Seldinger technique or the Trocar technique. The Seldinger technique is a multistep approach involving initial needle puncture, guidewire insertion, tract dilation, and subsequent catheter placement over the wire. In contrast, the Trocar functions as a single-step procedure, involving the direct insertion of the drainage catheter mounted on a sharp trocar assembly which may offer advantages in terms of reduced procedural time and technical simplicity [6]. Previous comparative studies [11, 12] indicate that the Trocar technique is as safe and effective as the Seldinger technique. These studies highlight that the Trocar technique is significantly faster and associated with reduced post-procedural pain. However, the choice of anatomical access route (transhepatic or transperitoneal) is a separate but related decision. The transperitoneal route is anatomically necessary in some patients but theoretically carries a higher risk of bile leakage because hepatic tissue tamponade is absent [13].

This risk is particularly elevated when utilizing the Seldinger technique, where sequential tract dilations can facilitate bile spillage into the peritoneal cavity, potentially exacerbating localized peritonitis and increasing post-procedural pain [11, 12].

Despite these findings, this study aims to evaluate the pre-specified interaction between insertion technique and anatomical access route on procedural time. The secondary outcomes are technical and clinical success, post-procedural pain, adverse events graded per SIR criteria, and 30-day mortality.

## Materials and methods

### Study population and design

This retrospective cohort study was conducted at Maharaj Nakorn Chiang Mai hospital between January 2020 and August 2024. The study protocol was approved by the Institutional Review Board (RAD-2567-6045), with a waiver of informed consent due to the retrospective nature of the study. We included consecutive patients aged > 15 years diagnosed with acute cholecystitis according to the Tokyo Guidelines 2018 (TG18) criteria [2] who underwent PC based on multidisciplinary consensus. Exclusion criteria were incomplete medical records or loss to follow-up.

Patients were excluded if they lacked essential data required for the primary analysis. These essential variables consisted of: (i) procedural time (start and end timestamps), (ii) the insertion technique used, (iii) the anatomical access route, (iv) a post-procedural analgesia record covering the first 24 h, and (v) 30-day clinical status. The detailed flow of patient inclusion and exclusion is illustrated in Fig. 2.

### Interventional procedures

PC procedures were performed by interventional radiologists with 3–20 years of experience, using either the Seldinger or Trocar technique under ultrasound guidance. A 6-Fr to 10-Fr locking pigtail drainage catheter was utilized. Local anesthesia (1% or 2% Lidocaine) was administered for all patients.

**Fig. 1:**
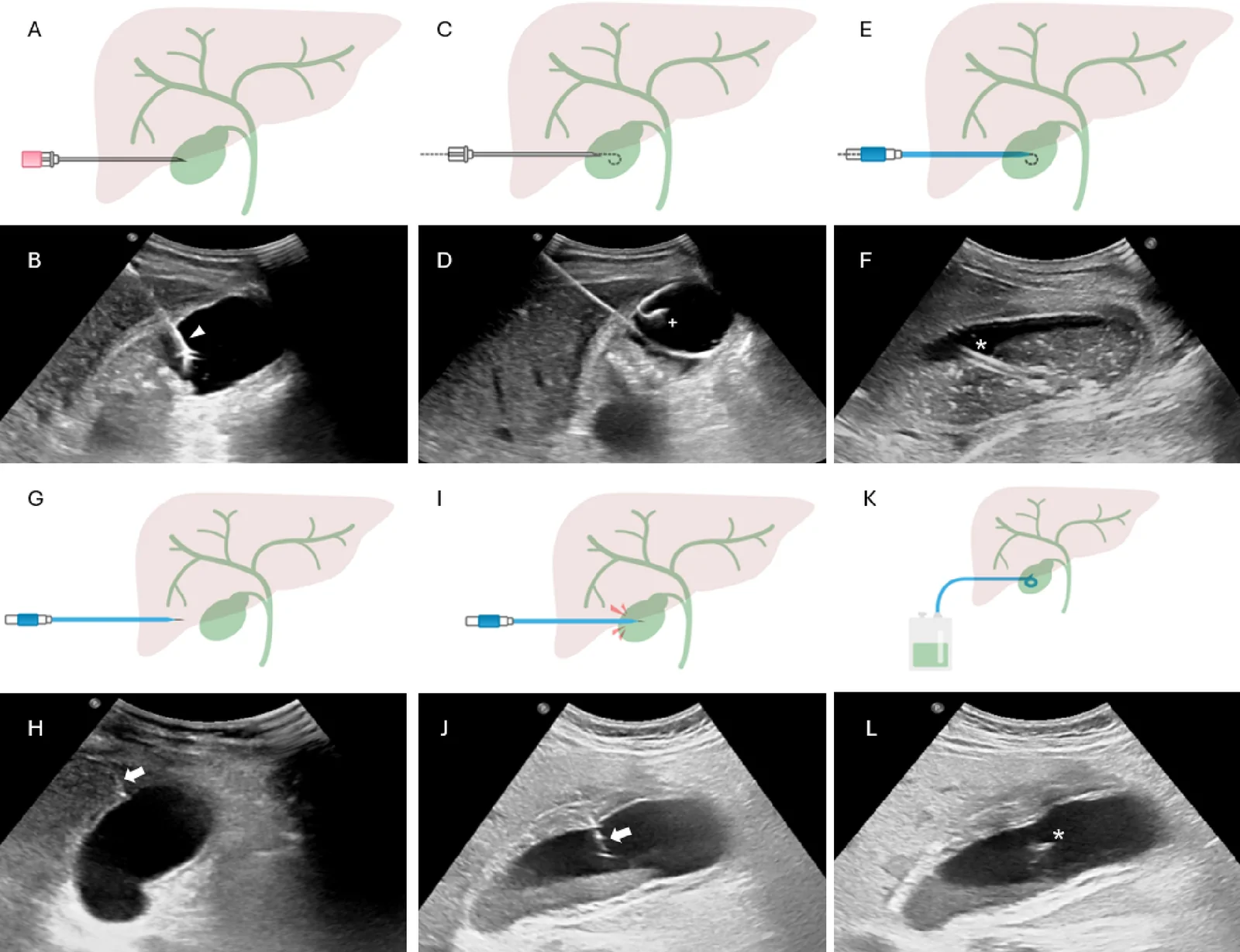
Procedural steps of percutaneous cholecystostomy (PC). Schematic diagrams and corresponding ultrasound images comparing the procedural steps of the Seldinger and Trocar techniques. The Seldinger Technique **A**–**F**: The procedure begins with an initial puncture using an 18-gauge needle (**A**,** B**). Upon verifying position, a 0.035-inch guidewire is inserted through the needle (**C**,** D**). Following tract dilation, the drainage catheter is advanced over the guidewire into the gallbladder (**E**,** F**). The Trocar Technique **G**–**J**: The catheter-trocar assembly is advanced to abut the gallbladder wall (**G**,** H**). A direct single-step puncture is then performed to insert the catheter into the lumen (**I**,** J**). Final Placement **K**,** L**: The procedure concludes with the pigtail catheter locked to form a loop securely within the gallbladder (**K**,** L**). Symbols: *Arrowhead*: Needle tip within the gallbladder lumen (Panel **B**). *Plus sign*: Echogenic line of the guidewire (Panel **D**). *Arrow*: Catheter-trocar assembly (Panels **H**, **J**). *Asterisk*: Drainage catheter shaft and pigtail loop (Panels **F**, **L**)

Seldinger Technique: This multistep approach involved initial gallbladder puncture with an 18-gauge needle. Upon bile aspiration to confirm intraluminal position, a 0.035-inch guidewire was advanced into the gallbladder. The tract was subjected to serial dilation to the appropriate caliber, followed by over-the-wire placement of the drainage catheter (Fig. 1, A–F).

Trocar Technique: This single-step procedure involved direct puncture using a pigtail drainage catheter mounted on a sharp central trocar (stylet) assembly, without prior guidewire placement. Once the catheter tip was visualized within the gallbladder lumen, the inner stylet was withdrawn while simultaneously advancing the catheter (Fig. 1, G–J).

The choice of technique, catheter size (6–10 Fr), and access route (transhepatic or transperitoneal) were determined at the operator’s discretion based on anatomical accessibility and patient characteristics. During the study window, the Seldinger technique was the historical default. The Trocar technique was adopted progressively from 2022 onward.

### Outcome definitions

Primary outcome; Procedural time defined as the duration from the administration of local anesthesia to the time when the tip of the catheter is located in the gallbladder lumen. In this retrospective study, procedural time was accurately calculated using the objective timestamps of the intra-procedural ultrasound images archived in our institution’s Picture Archiving and Communication System (PACS), measuring from the initial image capturing local anesthesia administration to the final image demonstrating the catheter looped within the gallbladder [14].

Secondary outcomes; Technical success defined as successful catheter placement within the gallbladder lumen yielding bile aspiration [6]. Clinical success defined as resolution of clinical symptoms (pain, fever) and improvement in the white-blood-cell count within 72 h after the procedure. Post procedural pain (strong-opioid requirement), given the retrospective design, post-procedural pain was assessed using a uniformly recorded objective surrogate for moderate-to-severe pain, defined as the requirement for strong opioid analgesics in the first 24 h. Our institutional pain-management protocol aligns with the WHO Pain Ladder guidelines, wherein strong (WHO Step-3) opioids (specifically parenteral morphine 2–4 mg IV, pethidine 25–50 mg IV, or fentanyl 25–100 µg IV) are strictly administered as rescue medication [15]. These analgesics are exclusively reserved for patients whose Numerical Rating Scale (NRS) score exceeds 4 and when Step-1 or Step-2 analgesics are judged inadequate. Although NRS scores were extracted from the medical records, these assessments were documented as part of routine clinical care rather than at standardized, pre-specified intervals. Because the exact timing of pain evaluations naturally varied among patients in this retrospective cohort, utilizing these scores as the primary pain outcome could introduce measurement bias. Therefore, the administration of strong opioids within the first 24 h was utilized as a more objective and uniformly recorded surrogate for severe pain. Adverse events were evaluated and classified according to the Society of Interventional Radiology (SIR) Adverse Event Classification [16]. For the purpose of this study, our safety analysis focused on major adverse events, specifically defined as ‘Severe’ (requiring substantial treatment, escalation in care, prolonged hospitalization) or ‘Life-threatening/Death’. 30-day mortality: vital status at 30 days post-procedure, with cause adjudicated by review of death certificates, blood and sputum cultures, microbiology, and post-procedural imaging where available. Cause-specific categories were procedure-related, uncontrolled non-biliary sepsis, and other causes.

### Data collection

Data collection was conducted by a radiology resident under the supervision of an experienced interventional radiologist by reviewing inpatient medical records and imaging studies (US and CT) via the Picture Archiving and Communication System (PACS).

The collected variables encompassed demographic and baseline characteristics, including gender, age, height, weight, BMI, underlying diseases, duration of symptom onset, and physical examination findings (e.g., palpable mass at RUQ). Diagnosis and disease severity were graded according to the Tokyo Guidelines 2018 (TG18). Additional recorded parameters included laboratory data (WBC count), clinical signs (fever, shock), and imaging findings (gallbladder dimensions, wall thickness, pericholecystic fluid and presence of gallstones). Regarding procedural details, the technique used (Seldinger vs. Trocar), drainage catheter size (6–8 Fr vs. 10 Fr), anatomical approach (transhepatic vs. transperitoneal), technical success, and procedural time were documented. Finally, outcomes were assessed based on clinical success, post-procedural pain (indicated by strong opioid requirement), 30-day mortality, and procedure-related complications (e.g., bile leak, bleeding, bowel injury).

### Statistical analysis

Continuous variables were reported as mean ± standard deviation (SD) for normally distributed data or median with interquartile range (IQR) for non-normally distributed data. Categorical variables were presented as frequencies and percentages. Between-group comparisons for continuous variables were performed using Student’s t-test for normally distributed data or the Mann-Whitney U test for non-normally distributed data. Fisher’s exact test was employed for comparisons of categorical variables. Standardized differences (STD) are reported alongside p-values to assess the magnitude of between-group differences; an STD < 0.10 indicates negligible imbalance, and an STD < 0.30 indicates acceptable balance.

For the primary outcome of procedural time, either multivariable Gaussian regression (for normally distributed data) or multivariable quantile regression (for non-normally distributed data) was used, with marginalization applied to estimate the marginal mean difference between treatment groups. This approach was selected because the marginalized mean is well-defined regardless of within-group skewness and remains interpretable on the original time scale. The model was adjusted for local signs of inflammation (Tokyo Guidelines diagnostic criteria), Tokyo severity grade, white blood cell count, presence of gallstones, significant comorbidities, catheter size, gallbladder length, gallbladder width, gallbladder wall thickness, and body mass index. For secondary outcomes of mortality rate and opioid use, multivariable logistic regression with marginalization was used to estimate marginal odds ratios between treatment groups, adjusting for the same covariates as the primary analysis. Subgroup analyses were performed to evaluate the interaction between the technique used and the anatomical approach (transhepatic vs. transperitoneal) regarding post-procedural pain. Sensitivity analyses were performed for all primary and secondary outcomes using confounder score and inverse probability weighting. The confounder score and Inverse probability weighting (IPW) method were constructed using the same covariates. All statistical analyses were conducted using Stata version 17 (StataCorp LLC, College Station, TX, USA).

## Results

### Study population & baseline characteristics

**Fig. 2:**
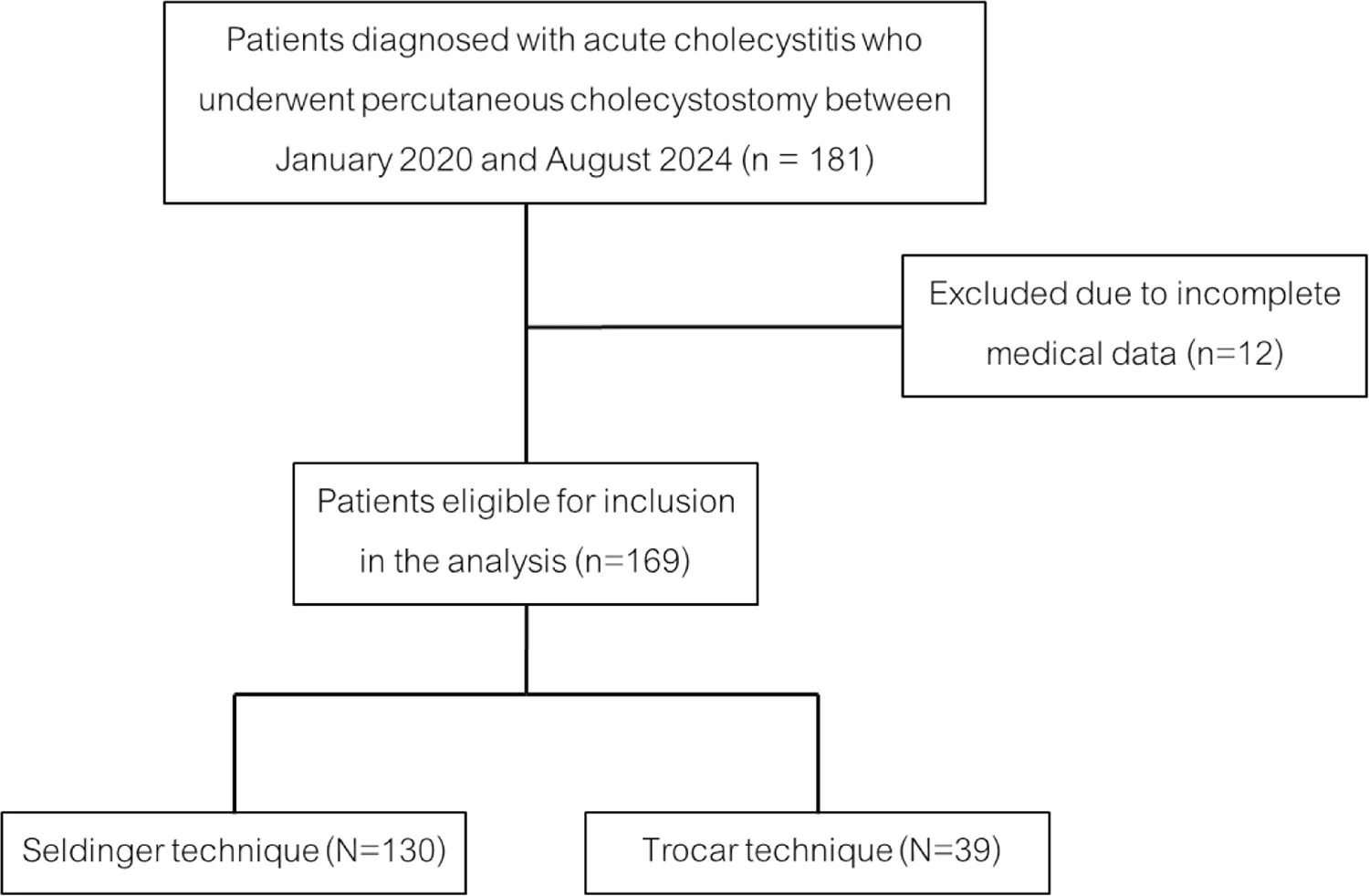
Study flow diagram

A total of 181 patients were initially enrolled in the study. Of these, 12 patients were excluded due to missing essential data. After applying the exclusion criteria, 169 patients remained eligible for inclusion in the final analysis: 130 in the Seldinger technique group and 39 in the Trocar technique group (Fig. 2). As detailed in Table 1, baseline demographics, including age, gender, BMI, and comorbidities, were comparable between groups. Disease severity markers, such as the TG18 severity grade and WBC counts, showed no statistically significant differences. Following inverse-probability-of-treatment weighting and confounder-score adjustment, standardized differences for all covariates remained below 0.3, indicating adequate balance between the study groups.

**Table 1:** Baseline characteristics

Characteristic	Seldinger (*N* = 130)	Trocar (*N* = 39)	*P*-Value	STD
Sex			0.578	0.1316
Male Female	75 (58%) 55 (42%)	25 (64%) 14 (36%)		
Age	70 (±16)	67 (±16)	0.272	0.2039
Height	160 (±9)	160 (±11)	0.888	0.0272
Weight	59 (±14)	64 (±21)	0.210	0.2531
BMI	23.0 (±5.0)	24.6 (±5.8)	0.139	0.2848
Duration of onset (Day)	2.00 (1.00, 3.00)	2.00 (1.00, 3.00)	0.446	0.1550
Diagnosis*			0.751	0.0617
Suspected Definitive	11 (8%) 119 (92%)	4 (10%) 35 (90%)		
Severity*			0.318	
Mild Moderate Severe	34 (26%) 69 (53%) 27 (21%)	6 (15%) 22 (56%) 11 (28%)		0.2678 0.0670 0.1736
White blood cell	15,614 (±7,547)	17,292 (±6,653)	0.185	0.2359
US parameter				
GB length	9.20 (8.30, 10.30)	9.50 (8.60, 11.10)	0.166	0.2487
GB width	3.75 (3.20, 4.40)	3.80 (3.40, 4.50)	0.781	0.0196
GB wall	0.50 (0.40, 0.60)	0.50 (0.40, 0.60)	0.206	0.1754
Murphy’s sign	119 (92%)	36 (92%)	>0.999	0.0282
GB fluid	54 (42%)	15 (38%)	0.853	0.0628
Gallstone	88 (68%)	29 (74%)	0.553	0.1474

*Diagnosis and severity according to Tokyo guideline 2018

*STD* Standardized difference, *GB* Gallbladder

### Procedural outcomes

Both techniques demonstrated high efficacy (Table 2). Technical success rates were 99.2% (129/130) for the Seldinger group and 100% (39/39) for the Trocar group (*p* > 0.99). Similarly, clinical success rates were comparable (98.5% vs. 94.9%, *p* = 0.23).

**Table 2:** Procedural outcomes and procedural time

Outcome	Seldinger (*n* = 130)	Trocar (*n* = 39)	Adjusted Difference/OR (95% CI)	*P*-value
Technical success, n (%)	129 (99.2)	39 (100)	–	> 0.99
Clinical success, n (%)	128 (98.5)	37 (94.9)	–	0.23
Procedural time (second), median (IQR)	246 (199–399)	81 (69–99)	−212.78 (−314.68, −110.88)†	< 0.001
Transhepatic approach	253 (207–339)	84.5 (68.8–94.5)	233.47 (88.08, 378.87)†	0.002
Transperitoneal approach	210 (179–224)	78 (73–95.5)	136.87 (81.34, 192.40)†	< 0.001

†Adjusted mean difference estimated from multivariable Gaussian regression

Procedural time reported as median (IQR)

Regarding procedural time, the Trocar technique was significantly faster. The median procedural time was 81 s (IQR: 69–99) for the Trocar group, compared to 246 s (IQR: 199–399) for the Seldinger group. Multivariable Gaussian regression confirmed that the Trocar technique was associated with a significant reduction in procedural time (adjusted mean difference: −212.78 s; 95% CI: −314.68, −110.88; *p* < 0.001). Sensitivity analyses were highly concordant, yielding an adjusted mean difference of −214.72 s (95% CI: −315.97, −113.48) by confounder-score adjustment, and −215.54 s (95% CI: −290.11, −140.98) by inverse probability weighting (IPW). Subgroup analyses by anatomical access route demonstrated that the Trocar technique remained significantly faster in both transhepatic and transperitoneal subgroups (Table 2).

### Post-procedural pain assessment

In the overall population, the proportion of patients requiring strong opioids did not differ significantly between the Seldinger and Trocar groups (29.2% vs. 17.9%; adjusted OR 1.01; 95% CI 0.47, 2.20; *p* = 0.97) (Table 3). Catheter size (10 Fr vs. <10 Fr) was not associated with the pain outcome (*p* = 0.59).

**Table 3:** Multivariable analysis of post-procedural pain (strong opioid requirement)

Subgroup	Seldinger (*n* = 130)	Trocar (*n* = 39)	Adjusted OR† (95% CI)	*P*-value
Overall population	38 (29.2%)	7 (17.9%)	1.01 (0.47–2.20)	0.973
Subgroup Analysis by route				
Transhepatic approach	33/117 (28.2%)	5/21 (23.8%)	0.97 (0.31–2.98)	0.952
Transperitoneal approach	5/13 (38.5%)	2/18 (11.1%)	7.36 (1.48–36.65)	0.015

†Adjusted for confounders including catheter size, comorbidities, and disease severity

However, subgroup analysis by anatomical route revealed a significant interaction. In the transhepatic subgroup, pain outcomes were similar between techniques (adjusted OR 0.97; 95% CI: 0.31, 2.98; *p* = 0.952). In contrast, within the transperitoneal subgroup, the Seldinger technique was associated with a significantly higher rate of strong opioid requirement compared to the Trocar technique (38.5% vs. 11.1%; adjusted OR 7.36; 95% CI: 1.48, 36.65; *p* = 0.015).

### Adverse events and mortality

Procedure-related adverse events were rare. One severe adverse event (0.8%) occurred in the Seldinger group, involving a cystic artery injury requiring embolization. No severe adverse event was observed in the Trocar group. Subgroup analysis for severe adverse events could not be performed due to complete separation resulting from data sparsity.

The 30-day mortality rate was higher in the Trocar group compared to the Seldinger group (23.1% vs. 6.2%; adjusted OR 0.16; *p* = 0.001). Subgroup analysis by access route showed this difference was directionally consistent in both transhepatic and transperitoneal subgroups (Table 4). However, a cause-specific mortality analysis suggested that this disparity was predominantly attributable to non-biliary causes. Specifically, the Trocar group had a significantly higher proportion of deaths attributed to uncontrolled sepsis (adjusted OR for Seldinger vs. Trocar: 0.04; 95% CI: 0.00, 0.67; *p* = 0.026). A review of death certificates and medical records confirmed that fatalities were primarily due to severe underlying conditions such as hospital-acquired pneumonia, infected aortic aneurysm, and refractory septic shock unrelated to the biliary procedure. There was no clinical or imaging evidence of biliary leakage or undrained collections in the peritoneum in this patient group.

**Table 4:** Cause-specific 30-day mortality analysis

	Seldinger (*n* = 130)	Trocar (*n* = 39)	Adjusted OR (95% CI)†	*P*-value
Total deaths	8 (6.2%)	9 (23.1%)	0.16 (0.05, 0.49)	0.001
By specific cause				
Procedure-related	0	0	–	–
Uncontrolled sepsis	1 (0.8%)	2 (5.1%)	0.04 (0.00,0.67)	0.026
Other cause‡	7 (5.4%)	7 (17.9%)	0.15 (0.05, 0.51)	0.002
Subgroup Analysis by route				
Transhepatic approach	7 (6%)	3 (15%)	0.26 (0.06, 1.23)	0.089
Transperitoneal approach	1 (7.7%)	6 (31.6%)	NA*	NA*

†Comparison of Seldinger vs. Trocar (Reference group)

‡Includes pneumonia, infected aortic aneurysm and myocardial infarction

*Adjusted OR could not be estimated due to complete separation

## Discussion

The present study demonstrates that both the Seldinger and Trocar techniques are highly effective for gallbladder decompression in patients with acute cholecystitis, achieving comparable technical and clinical success rates exceeding 95% [7, 17]. However, our analysis highlights two distinct advantages of the Trocar technique: a significantly shorter procedural time and a favorable pain profile specifically in patients requiring a transperitoneal approach. Furthermore, while a higher crude mortality rate was observed in the Trocar group, a cause-specific analysis suggested that these fatalities were predominantly attributed to underlying non-biliary sepsis.

Regarding procedural time, the Trocar technique was significantly faster, reducing the median operative time by approximately 3 min compared to the Seldinger technique (81 vs. 246 s). This finding is consistent with previous randomized trials by Reppas et al. [11] and Arkoudis et al. [12], which attributed the speed of the Trocar technique to its single-step nature, eliminating the need for guidewire manipulation and serial tract dilation. In critically ill patients with hemodynamic instability, this reduction in procedural time is clinically meaningful, potentially minimizing the duration of uncomfortable positioning and physiological stress. Importantly, the magnitude of the procedural-time difference was concordant across the primary regression, confounder-score adjustment, and IPW analyses, supporting the robustness of this finding to measured confounders.

A novel finding of this study is the significant interaction between the insertion technique and the anatomical approach regarding post-procedural pain. While the overall opioid requirement was similar between groups, subgroup analysis revealed that the Seldinger technique was associated with a 7-fold increased risk of severe pain (strong opioid requirement) when performed via the transperitoneal route, whereas no such difference existed in the transhepatic subgroup. This observation challenges the traditional view that the Seldinger technique is inherently less traumatic due to the use of a smaller initial needle [18].

We hypothesize that the mechanism underlying this pain discrepancy involves bile leakage and subsequent localized peritonitis [19]. In the transhepatic approach, the liver parenchyma provides a natural tamponade effect, sealing the tract created by serial dilation [20]. However, in the transperitoneal approach, the gallbladder fundus is directly punctured through the peritoneum without intervening liver tissue. The multistep nature of the Seldinger technique inherently carries a risk of bile spillage, particularly during instrument exchanges. Specifically, when a dilator is withdrawn to be replaced by the drainage catheter, a transient gap is created between the puncture site on the gallbladder wall and the indwelling guidewire. This momentary lack of tamponade allows bile to leak into the peritoneal cavity and irritate the sensitive parietal peritoneum before the catheter is secured. In contrast, the Trocar technique utilizes a pre-loaded catheter that immediately tamponades the puncture site upon entry, theoretically minimizing bile spillage. Therefore, our data suggests that the Trocar technique may be associated with reduced post-procedural pain when transperitoneal access is anatomically unavoidable.

Regarding safety, the Trocar group exhibited a higher 30-day mortality rate compared to the Seldinger group (23.1% vs. 6.2%). However, this statistical difference warrants careful interpretation. Our cause-specific mortality analysis suggested the disparity was predominantly attributable to non-biliary causes, such as hospital-acquired pneumonia and refractory septic shock from other sources. No procedure-related deaths were observed in either group. This pattern suggests a degree of selection bias inherent to retrospective practice: interventional radiologists may have preferentially selected the quicker Trocar technique for “moribund” or biologically unstable patients who required immediate source control but had a poor overall prognosis regardless of the intervention. Thus, the higher mortality likely reflects the baseline gravity of the patient’s condition rather than the safety of the technique itself.

This study has several limitations. First, its retrospective nature and the imbalance in sample size between groups (Seldinger *n* = 130 vs. Trocar *n* = 39) may introduce selection bias. Although rigorous statistical adjustments were utilized to account for observable covariates, the potential impact of unmeasured confounders remains significant, particularly regarding clinical decision-making for technique selection in highly unstable patients. Second, post-procedural pain was assessed using opioid requirements as a surrogate marker rather than subjective pain scores (VAS). While this metric provides a more uniform objective reflection of severe pain, we acknowledge its inherent limitations, as it cannot perfectly capture subjective pain experiences and may be influenced by clinician variability and prescribing practices. Third, the sample size within the transperitoneal subgroup was relatively small. Given the wide confidence interval, the strength of this conclusion should be carefully balanced against the limited sample size, and these findings must be interpreted with caution to avoid overinterpretation.

In conclusion, the Trocar technique is a time-efficient alternative to the Seldinger technique for percutaneous cholecystostomy, with comparable technical and clinical success. Our findings suggest that, when transperitoneal access is anatomically unavoidable, the Trocar technique may be preferred, as it is associated with shorter procedural time and a lower risk of severe post-procedural pain. The technique-by-route interaction observed here should be confirmed in larger prospective studies before it is applied as a standard recommendation.

## Data Availability

The datasets generated and analyzed during the current study are not publicly available due to patient privacy and institutional data governance policies, but are available from the corresponding author on reasonable request.
